# Nitrilase 1 modulates lung tumor progression *in vitro* and *in vivo*

**DOI:** 10.18632/oncotarget.7820

**Published:** 2016-03-10

**Authors:** Yong Antican Wang, Yunguang Sun, Justin M. Le Blanc, Charalambos Solomides, Tingting Zhan, Bo Lu

**Affiliations:** ^1^ Department of Radiation Oncology, Thomas Jefferson University, Philadelphia, PA, 19107, USA; ^2^ Department of Cancer Biology, Thomas Jefferson University, Philadelphia, PA, 19107, USA; ^3^ Department of Pathology, Thomas Jefferson University, Philadelphia, PA, 19107, USA; ^4^ Department of Pharmacology, Thomas Jefferson University, Philadelphia, PA, 19107, USA; ^5^ Department of Pathology, Medical College of Wisconsin, Milwaukee, WI, 53226, USA

**Keywords:** NIT1, lung cancer, KRAS, NSCLC, tumor suppressor

## Abstract

Uncovering novel growth modulators for non-small cell lung cancer (NSCLC) may lead to new therapies for these patients. Previous studies suggest Nit1 suppresses chemically induced carcinogenesis of the foregut in a mouse model. In this study we aimed to determine the role of Nit1 in a transgenic mouse lung cancer model driven by a G12D Kras mutation. Nit1 knockout mice (Nit1^−/−^) were crossed with Kras^G12D/+^ mice to investigate whether a G12D Kras mutation and Nit1 inactivation interact to promote or inhibit the development of NSCLC. We found that lung tumorigenesis was suppressed in the Nit1-null background (Nit1^−/−^:Kras^G12D/+^). Micro-CT scans and gross tumor measurements demonstrated a 5-fold reduction in total tumor volumes compared to Nit1^+/+^Kras^G12D/+^ (p<0.01). Furthermore, we found that Nit1 is highly expressed in human lung cancer tissues and cell lines and use of siRNA against Nit1 decreased overall cell survival of lung cancer cells in culture. In addition, cisplatin response was enhanced in human lung cancer cells when Nit1 was knocked down and Nit1^−/−^:Kras^G12D/+^ tumors showed increased sensitivity to cisplatin *in vivo*. Together, our data indicate that Nit1 may play a supportive role in the modulation of lung tumorigenesis and represent a novel target for NSCLCs treatment.

## INTRODUCTION

Lung cancer is the leading cause of cancer mortality worldwide [1, 2]. It generally presents with genomic instability and abnormalities that researchers have exploited as novel sources for targeted therapeutics [3, 4]. The success of these research efforts in utilizing targeted therapeutics has aided clinicians in the management of lung cancer patients [5–7]. Non-small cell lung cancers (NSCLCs), which represent 85% of all lung cancers, generally present at very advanced stages requiring intensive and multimodal treatments [8]. Kras mutations, present in about 20-30% of NSCLCs, lead to the over activation of the mitogen-activated protein kinase (MAPK) pathway, which has diverse impacts on cellular proliferation [9]. Mutations in the Kras pathway have continually led cancer patients to decreased responses to chemotherapeutics and radiation therapy treatments [10]. The overall decreased response rates due to activating mutations in NSCLCs make it critically important to find new targets.

Galperin and Koonin listed the ‘top 10’ attractive targets with the expectation that characterization of these conserved proteins would reveal new aspects of biology. The fourth entry in the ‘top 10’ table was Nitrilase 1 (Nit1), a gene conserved from bacteria, yeast, plants, insects, invertebrates to vertebrates and mammals [11].

The nitrilase superfamily consists of thiol enzymes involved in natural product biosynthesis and post-translational modification in plants, animals, fungi and prokaryotes. 9/13 branches have known or deduced specificity for specific nitrile- or amide-hydrolysis or amide-condensation reactions [12]. In Arabidopsis, there are four nitrilases (Nitrilase 1–4), of which Nit1 shows the highest homology to animal and bacterial nitrilases. It was found that in plants, Nit1 is required to repress proliferation and in its absence causes cells to become polyploidy due to defects that occur in cytokinesis [13]. All mammalian species contain Nit1 genes, which share ~40% sequence homology; however, the substrate of Nit1 still has not been discovered. Recent studies of Nit1 have begun to indicate its potential role in tumorigenesis. Although Nit1-deficient mice have a normal life cycle, reproduction, and do not develop spontaneous tumors, they are more sensitive to carcinogen-induced forestomach tumors [14, 15]; in addition, ectopic expression of Nit1 leads to caspase activation and apoptosis, and may play a role in DNA damage-induced apoptosis in cell models [14]. The culmination of these data led us to investigate the role that Nit1 may have in modulation of lung cancer.

## RESULTS

### Generation and identification of the Nit1^−/−^ Kras^G12D/+^ mouse lung cancer model

**Figure 1:**
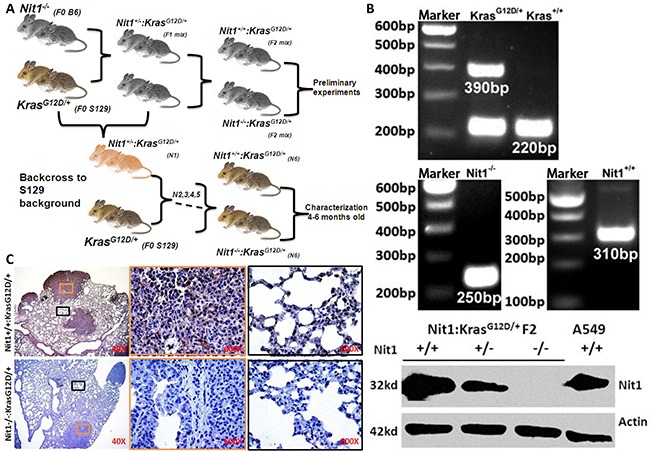
Generation and identification of the Nit1^−/−^:Kras^G12D/+^ mouse lung cancer model **A.** Mouse breeding strategy. **B.** Mouse genotyping and western blot of Nit1 in F2 mice of various genotypes. Nit1 was highly expressed in wild-type and human lung cancer cell line (A549), lower expressed in Nit1^+/−^ Kras^G12D/+^ and absent in Nit1^−/−^ Kras^G12D/+^ mouse, actin expression was also analyzed as a control. **C.** Immunohistochemistry of Nit1 in mouse lung tissue sections from two genotypes. There is no signal of Nit1 in Nit1^−/−^:Kras^G12D/+^ mouse (lower panel) while Nit1 is positive in Nit1^+/+^:Kras^G12D/+^ mouse lung tissue (top panel). High magnification of tumor (orange) and normal (black) lung tissues was photographed.

To investigate the impact of Nit1 on lung carcinogenesis and progression, we crossed Nit1^−/−^ mice with Kras^G12D/+^ spontaneous lung adenocarcinomas mice as outlined in Figure 1A. PCR-based genotyping established the genetic status of Nit1 and Kras^G12D^ and Western blots of lung tissue lysate confirmed the deficiency of Nit1 protein as shown in Figure 1B. Immunohistochemistry also demonstrated a lack of Nit1 expression in knockout strains in both tumor and the surrounding lung tissues, while positive staining of Nit1 can be found in the nucleus and cytoplasm of wild-type mice lung tissue cells (Figure 1C), which is consistent with Huebner's data [14] and the Human Protein Atlas (HPA) Nit1 datasets [http://www.proteinatlas.org/ENSG00000158793-NIT1/tissue/lung].

### Tumor burden and survival proportion of Nit1 deficient Kras^G12D/+^ mouse lung cancer model

**Figure 2:**
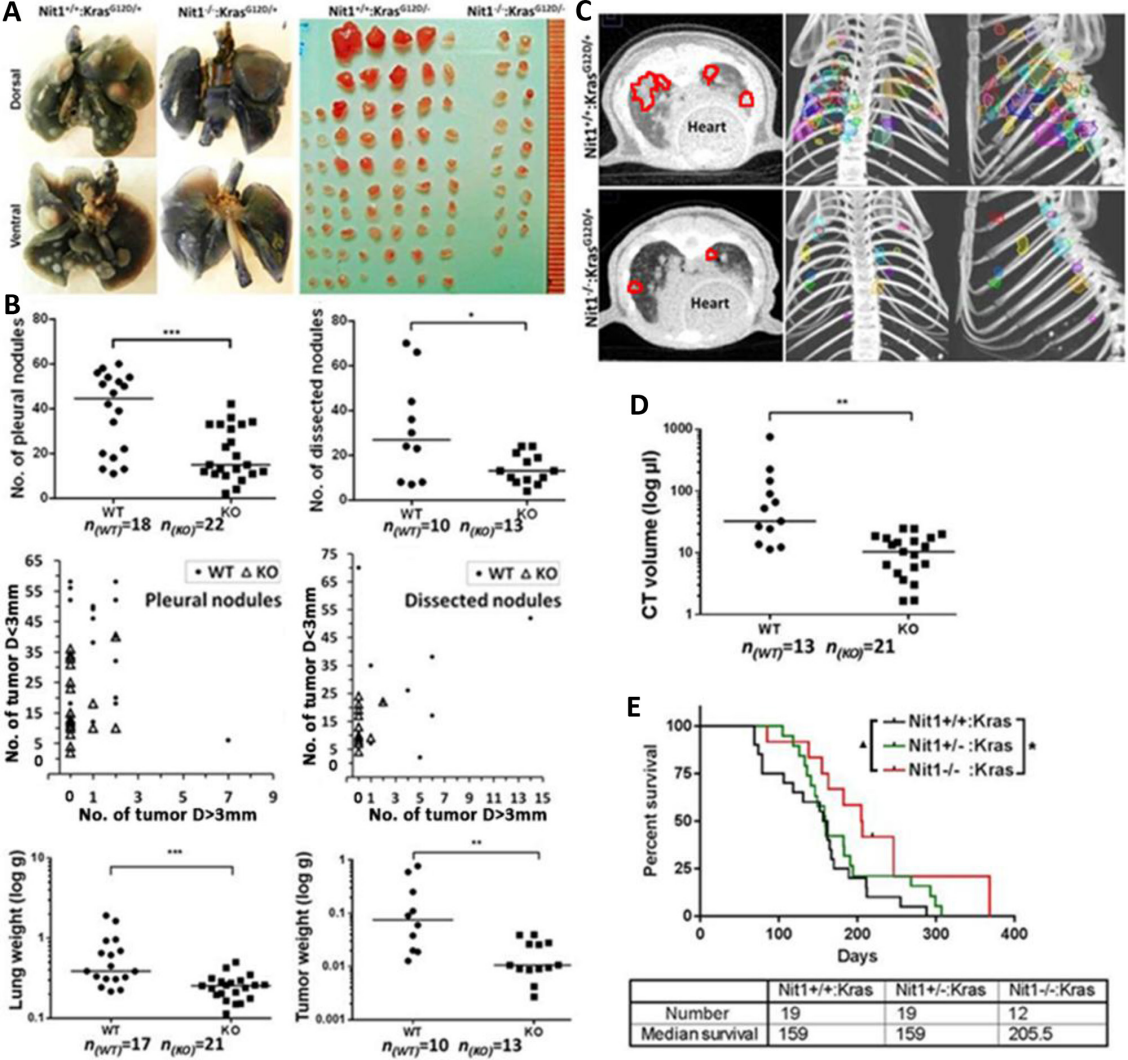
Tumor burden and survival proportion of Nit1 deficient Kras^G12D/+^ mouse lung cancer model **A.** Representative images of 6 months old mice lung tumor burden. Left: Ink staining showing tumor nodules at the pleural surface. Right: tumor nodules dissected from tumor-bearing lungs. **B.** Statistical analysis of number of all pleural tumor nodules (top left) or dissected tumors (top right); size distribution of pleural tumor nodules (>3mm vs. <3mm) (mid left), dissected tumor nodules (mid right). Each dot/triangle in these two plots represents total tumors of one mouse, instead of a tumor sample. The x y co-ordinates show the number of surface/dissected nodules diameter D> 3mm (horizontal) and the number of D< 3mm (vertical) of each mouse; weight of tumor-bearing lungs (bottom left) or dissected tumors (bottom right) from Nit1^+/+^:Kras^G12D/+^ (WT) or Nit1^−/−^:Kras^G12D/+^ (KO) mice. Mann Whitney test, * P<0.05, ** P<0.01, *** P<0.001; n: number of mice. **C.** Representative micro-CT image. Left: Axial CT images, tumor area are marked with red circles; Right: color-rendered 3D representations of tumor volumes from coronal and sagittal planes. **D.** Statistical analysis of tumor volumes measured through micro-CT. Mann Whitney test, ** P<0.01. **E.** Survival proportions of Nit1 deficient Kras^G12D/+^ mice models. Log-rank (Mantel-Cox) test for survival curves * P<0.05, and Log- rank test for trend ▲ P<0.05.

To determine the lung tumor burden of Nit1^+/+^:Kras^G12D/+^ vs. Nit1^−/−^:Kras^G12D/+^, mice were sacrificed when they reached 4-6 months of age. Tumor burden was measured through a number of approaches. First, we counted and graphed the lung tumor nodules at the pleural surface, and then nodules dissected from the tumor-bearing lungs bigger than 1mm in diameter were counted and graphed. As shown in Figure 2A and 2B top, the number of pleural (p<0.001) or dissected tumor nodules (p<0.05) was significantly reduced in the Nit1^−/−^:Kras^G12D/+^ mice in comparison to Nit1^+/+^:Kras^G12D/+^ mice; in addition, the size of tumor nodules are much smaller in Nit1^−/−^:Kras^G12D/+^ mice (Figure 2A, 2B middle). Next, we measured the weight of tumor-bearing lungs as well as dissected tumors from mice of the two genotypes and demonstrated that Nit1^+/+^:Kras^G12D/+^ mice have a heavier tumor load. Because micro-CT is an effective tool to noninvasively measure the growth of primary lung cancers and assess the tumor therapeutic response in genetically engineered mice [16], we contoured the tumor nodules and calculated tumor volumes through the 3D reconstructed micro-CT images using MIM software (Cleveland, OH, USA) [17]. As shown in Figure 2C, Nit1^−/−^:Kras^G12D/+^ mice have fewer and smaller lung lesions on CT with a 5-fold reduction of total tumor volume (median) compared with Nit1^+/+^:Kras^G12D/+^ mice (Figure 2D, p<0.01). Figure 2E shows the effect of Nit1 deficiency on survival of Kras^G12D/+^ mice. Remarkably, Nit1 deficient mice have a trend to live longer (Log-rank test for trend, ▲ P<0.05) though the median survival has no difference in Nit1^+/−^:Kras^G12D/+^ vs Nit1^+/+^:Kras^G12D/+^, however, Nit1^−/−^:Kras^G12D/+^ mice have significantly elongated life spans when compared with wild-type controls (Mantel-Cox test, * P<0.05).

### Reduced tumor lesions in Nit1 deficient Kras^G12D/+^ mice

**Figure 3:**
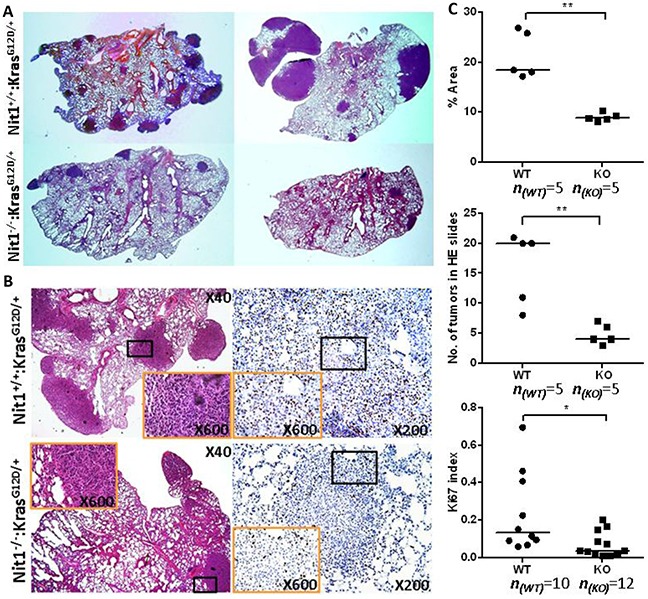
Reduced tumor lesions in Nit1 deficient Kras^G12D/+^ mice **A.** Representative H&E whole-slide scan images shown tumor number and area difference. **B.** Representative pathologic micrographs of the lung tumor lesions by H&E (left) and Ki67 IHC staining (right). Orange rectangle is the higher magnification of lower magnification (black) lung tumor area. **C.** Statistical analysis of tumor lesions in three sections from each mouse (identical levels, mean values), tumor area (top), number of total nodules (middle) and Ki67-positive cells (bottom) in similar size tumors, Mann Whitney test, * P<0.05, ** P<0.01.

H&E staining of Nit1^+/+^:Kras^G12D/+^ vs. Nit1^−/−^:Kras^G12D/+^ mice lung lesions were analyzed by a pathologist who specializes in lung cancer. These lung lesions obtained from both groups were pathologically indistinguishable adenocarcinoma (Figure 3B left) and the final tumor incidences of both genotypes are similar (100% at 4-6 months old). Cross-sections of tumor-bearing lungs following H&E staining showed Nit1^−/−^:Kras^G12D/+^ mice have decreased tumor area (Figure 3A, 3C top) and or decreased tumor number (Figure 3A, 3C middle) compared with wild-type controls. IHC staining of proliferation marker Ki67 indicated that tumor proliferation was suppressed significantly in Nit1^−/−^:Kras^G12D/+^ mice (Figure 3B, 3C bottom).

### Increased NIT1 expression in human lung cancer

**Figure 4:**
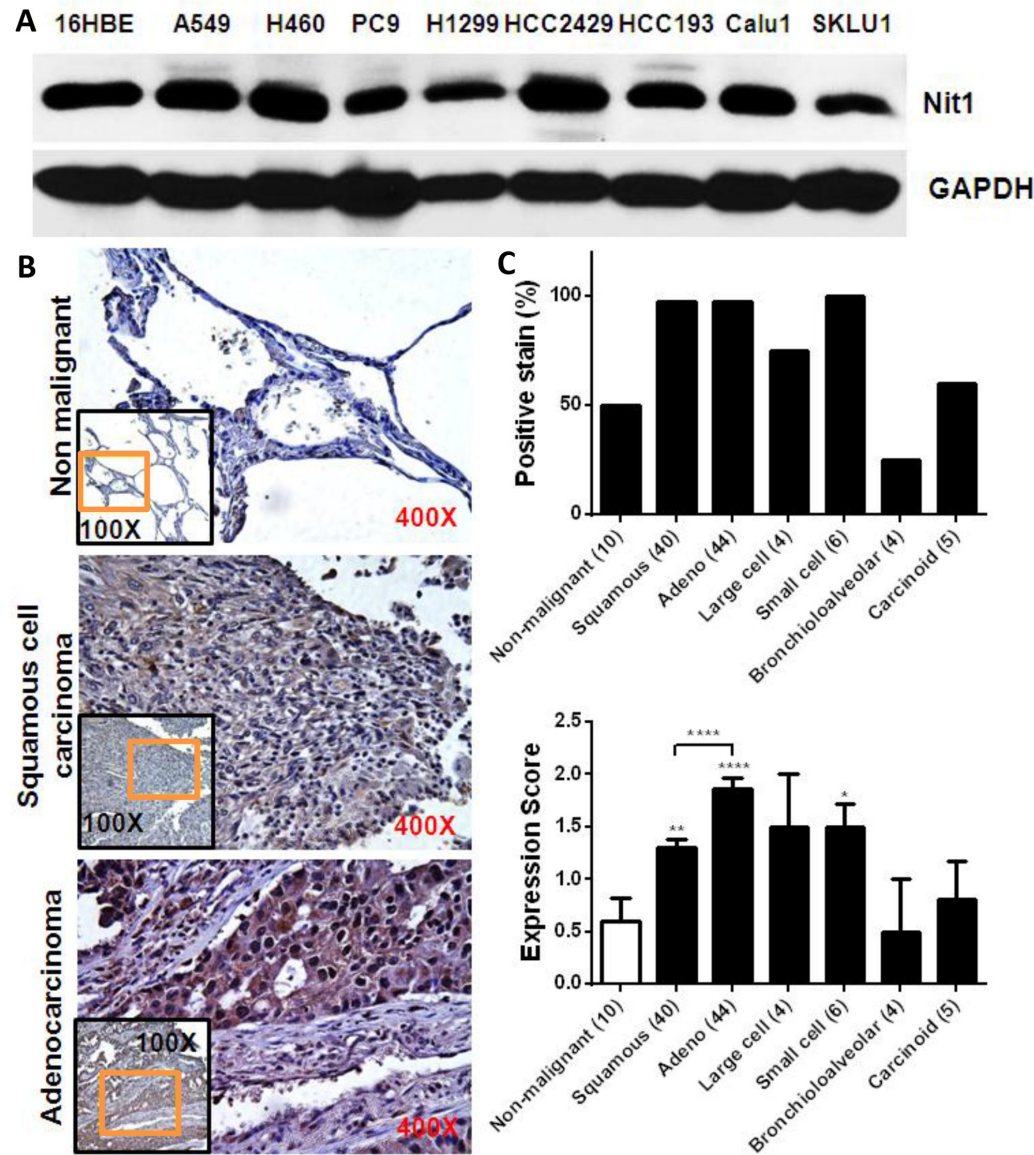
Increased Nit1 expression in human lung cancer **A.** Nit1 expression in human immortalized bronchial epithelia (16HBE) and NSCLCs cell lines. **B.** Representative examples of IHC staining of Nit1 in non-malignant lung tissue and tumors as indicated. Higher magnification of orange rectangle area in the lower magnification black rectangle is shown as background image. **C.** IHC staining of Nit1 in lung cancer samples was quantitated for percentage of positive Nit1 staining and their expression scores for each subtype of lung cancer. Data is shown as mean ±SEM for Nit1 expression score, Mann Whitney test, * P<0.05, ** P<0.01, **** P<0.0001.

Western blots confirmed Nit1 expression in immortalized human bronchial epithelial cells and multiple human NSCLCs cell lines (Figure 4A). We then performed immunohistochemistry staining on a human lung cancer tissues (TMA) slide. Representative examples of IHC for Nit1 in squamous cell carcinoma and adenocarcinoma were shown in Figure 4B lower panels. In the 5/10 non-malignant lung tissues (from patients with non-malignant lung diseases), weakly Nit1 staining (expression score ~0.6) can be identified within the pneumocytes and macrophages (Figure 4B top, 4C lower). However, positive staining of Nit1 in lung cancer cells is more prevalent especially in squamous cell carcinoma (97.5%, Figure 4B middle, 4C upper) and adenocarcinoma (97.7%, Figure 4B bottom, 4C upper). Quantitation of the Nit1 staining amongst distinct human lung tumors demonstrated an approximately 2-fold increase of Nit1 staining intensity in squamous cell carcinoma (p<0.01) and 3-fold increase in adenocarcinoma (p<0.0001) over non-malignant lung tissues (Figure 4C lower).

### Nit1 knockdown decreases survival of multiple human lung cancer cell lines

**Figure 5:**
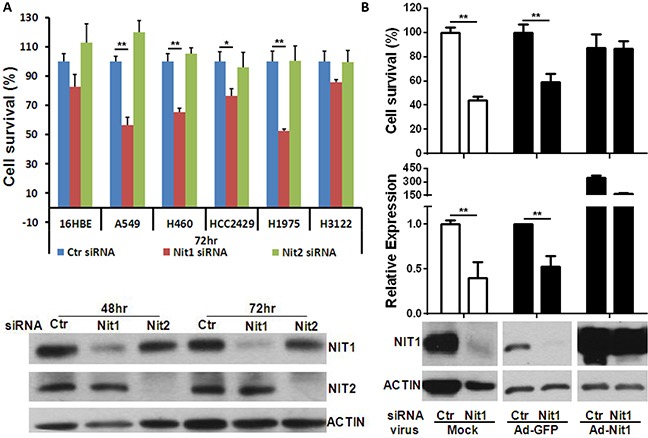
Nit1 knockdown decreases survival of multiple human lung cancer cell lines **A.** Cell survival post Nit1/Nit2 siRNA in multiple human lung cancer cell lines. Upper panel shown: Cell viability measured by MTS assay following various lung cancer cell lines transfected by control siRNA, siRNAs against Nit1 or Nit2, t-test, * P<0.05, ** P<0.01. Lower panel: western blot confirms knockdown of Nit1 or Nit2 by their siRNA in A549 cells. **B.** Nit1 siRNA knockdown with or without the rescue by Nit1-overexpressing adenovirus in A549 cells. Adenovirus MOI: 1:20, Incubation time: 72hr post siRNA transfection. Upper panel: Cell survival inhibition was rescued by over-expression of Nit1, in comparison to GFP overexpression (Ad-GFP), t-test, * P<0.05, ** P<0.01; middle panel: Relative expression level of Nit1 mRNA (normalized to mRNA level of GAPDH); Lower panel: western blot showing NIT1 protein level following transfection of Nit1 siRNA with or without Nit1-overexpressing adenovirus.

To determine whether lung cancer is dependent upon a high level of Nit1 expression, we tested lung cancer cell survival following Nit1 knockdown through siRNA using a specific siRNA smart pool. BLAST searches and scramble siRNA pools that do not match any known mammalian GENEBANK sequences determined the sequence specificity. In addition, we used siRNA against Nit2 as a gene control because Nit2, like Nit1, belongs to the nitrilase superfamily and structurally clustered with Nit1 in Arabidopsis which shares 55% homology of Nit1 [18]. Knocking down of Nit1 or Nit2 in A549 lung cancer cells were validated by Western blotting as shown in the lower panel of Figure 5A. The upper panel of Figure 5A demonstrates that the survival reduction of most of the tested lung cancer cell lines is only observed in cells transfected with siRNA against Nit1, but not in cells transfected with control siRNA or siRNA against Nit2. To further confirm that the survival reduction was due to Nit1 knockdown specifically, Nit1 overexpressing adenovirus was used to restore Nit1 level post knockdown. Quantitative PCR and Western blot analysis confirmed that Nit1 mRNA and protein levels were decreased after Nit1 siRNA and then restored to a higher level compared with adenovirus GFP control groups. Cell survival was rescued upon Nit1 overexpression, which implies that reduction of lung cancer survival depends on Nit1 level, as shown in Figure 5B.

### Nit1 deficiency enhances therapeutic effect of cisplatin to NSCLCs

**Figure 6:**
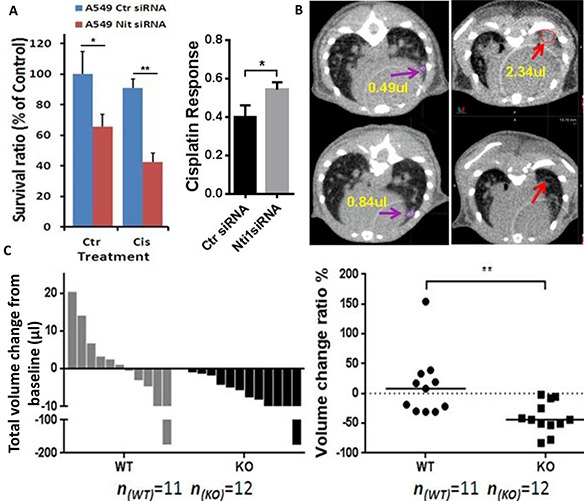
Nit1 deficiency enhances therapeutic effect of cisplatin to NSCLCs **A.** Knockdown of Nit1 increases cisplatin response in A549 cells. MTS cell survival assay, 48h after 10μM cisplatin and post 24h after siRNA against Nit1. t-test, * P<0.05, ** P<0.01 **B.** Robust cisplatin response in Kras^G12D/+^ mice without Nit1. After 4 doses of cisplatin treatment at 7.5 mg/kg every 3 days, representative micro-CT axial images shown tumor growth in Nit1^+/+^:Kras^G12D/+^ mouse (left, purple arrows with yellow value of volumes); tumor vanished in Nit1^−/−^:Kras^G12D/+^ mouse (right, red arrows with yellow value of volumes). **C.** Statistical analysis of cisplatin response. Waterfall plot of total volume change before and after treatment (left), each bar represents one individual mouse; Relative volume change ratio or drug response (Right), each dot represents one individual mouse. Mann Whitney test, ** P=0.002. WT: Nit1^+/+^:Kras^G12D/+^; KO: Nit^1−/−^:Kras^G12D/+^.

To determine the impact of Nit1 in mediating therapeutic effects against lung cancer, we knocked down Nit1 through siRNA and followed with cisplatin treatment on the relatively cisplatin resistant human lung cancer cell line A549. As shown in Figure 6A, there was no significant decrease in cell survival after cisplatin treatment in Nit1 wild type (100% vs. 90%, p>0.05, left blue bars), while it was significantly decreased with Nit1 knockdown (60% vs. 40%, p<0.05, red bars). And the cisplatin response after Nit1 knockdown was remarkably increased (p<0.05, Figure 6A right). Reductions in cell survival indicate that Nit1 deficiency might sensitize cisplatin response in human lung cancer. To confirm this finding *in vivo*, we compared the cisplatin responses in Nit1^−/−^:Kras^G12D/+^ mice with their wild-type counterpart, since Kras^G12D/+^ mice easily acquire cisplatin resistance. Before and after 4 doses of cisplatin treatment at 7.5 mg/kg every 3 days, tumor volumes were measured by the micro-CT. All tumor-bearing Nit1^−/−^:Kras^G12D/+^ mice demonstrated tumor shrinkage, whereas more than half of the Nit1 wild-type group had persistent tumor growth as shown by the waterfall plot (Figure 6C). Representative CT images of tumor volume increasing in Nit1^+/+^:Kras^G12D/+^ mice (purple arrows), while decreasing in Nit1^−/−^:Kras^G12D/+^ mice (red arrows) were shown in Figure 6B. The observed difference is statistically significant (p<0.01 Figure 6C).

## DISCUSSION

Increasing knowledge of activating driver mutations in NSCLCs has prompted the development of various targeted therapeutics [19, 20]. Studies have indicated that activating mutations such as Kras mutations are poor prognostic indicators and predictors for poor response to standard therapies [7, 21–23]. Based on these findings, it is clear that novel therapeutic targets are urgently needed to improve tumor sensitivities.

Nit1 was initially identified as a gene fused to Fhit in Drosophila melanogaster and Caenorhabditis elegans [24]. Based on the ‘Rosetta stone’ hypothesis [25], previous studies predicted Fhit and Nit1 would function in the same pathway in mammals and described Nit1 as possessing tumor suppressor characteristics in mice forestomach tissue and additive to gene FHIT [15]. However, the physical interaction between Fhit and Nit1, which are located on two different chromosomes in mammals, has not been confirmed [26]. In addition, web-databases (GeneCards, Ensembl et al.) show NIT1 copy number and mRNA levels are increased in human lung cancer samples compared with normal tissue, while there is no FHIT protein expression in human (Oncomine, COSMIC) or mouse lung tissue [14]. All these information suggest that gene NIT1 may function independently and may not be suppressor in human lung cancer.

To investigate whether Nit1 deficiency may promote or inhibit the development of NSCLCs, we knocked out Nit1 in Kras^G12D/+^ mice lung cancer model background (Figure 1). Surprisingly, Nit1^−/−^:Kras^G12D/+^ mice showed significantly decreased tumor burden (lesions, volume, lung and tumor weight) and significant life extension compared with Nit1^+/+^:Kras^G12D/+^ (Figure 2 and 3).

We confirmed Nit1 expression in multiple human NSCLCs cancer cells and investigated the relevance of Nit1 to human lung cancers through TMAs. The relative abundance of Nit1 was increased 2- to 3-fold in NSCLCs compared to non-malignant lung tissues, with adenocarcinomas having the highest Nit1 immune-staining intensity (Figure 4). Our findings are consistent with the datasets of Oncomine, so we further investigated lung cancer cell survival post gene NIT1 knock down. Our data showed that knocking down NIT1, but not NIT2, can decrease cell viability in various lung cancer cell lines, and that it can be rescued after NIT1 expression levels were restored with recombinant human Nit1 adenovirus. Increased Nit1 abundance in NSCLCs and Nit1 dependent lung cancer cell survival implied that the NIT1 gene could not be a suppressor in cultured human lung cancer cell lines. Based on our data and previous studies there appears to be an optimal level of Nit1 required for appropriate cellular proliferation. Either overexpression or knockdown could jeopardize the survival of cells; while their biological results might be depend on tissue or cell types.

Since gene mutations of the Kras pathway lead cancer patients to decreased responses to therapy treatments, we investigated the impact of Nit1 in mediating therapeutic effects against lung cancer. As shown in Figure 6A, significantly reduced cell survival post Nit1 knockdown combined with cisplatin treatment in resistant A549 indicated that Nit1 deficiency may sensitize cisplatin response in human lung cancer. Cisplatin treatment on Kras^G12D/+^ mice confirmed this finding *in vivo*. We tracked tumor nodules by the micro-CT before and after cisplatin treatment. The total tumor volume was decreased in some Nit1^+/+^:Kras^G12D/+^ mice, but more than half of them had persistent tumor growth, whereas all Nit1^−/−^:Kras^G12D/+^ mice demonstrated significant tumor shrinkage (Figure 6B, 6C).

Despite the early descriptions of Nit1 potentially functioning like a tumor suppressor when overexpressed [14], our data showed that Nit1 knockdown could decrease cell viability in various lung cancer cell lines and lung tumorigenesis was significantly suppressed in Nit1 knockout Kras^G12D/+^ mice. In addition, Nit1 knockdown has the ability to sensitize NSCLCs to cytotoxic agents (e.g. cisplatin). Based on these data, Nit1 may modulate growth patterns in lung cancer cells and maybe a new target for NSCLCs treatment in future. Similar to the results we demonstrated, Zheng et al. recently showed that knockdown of Nit2 in colon cancer produced decreased levels of cellular proliferation. Their data indicated that Nit2, a member of the nitrilase protein family, which also has been reported as a suppressor [27], acts as an oncogene in human colon cancer [28]. Together these data appear to implicate the nitrilase protein family as interesting targets for future research on tumorigenesis and mechanisms of action.

In the present study, we have shown the impact of knocking down Nit1 *in vivo* and *in vitro* in NSCLCs. We found reduced tumor lesions in Nit1 deficient Kras^G12D/+^ mice and then confirmed that human NSCLCs have high levels of Nit1. In addition, deficiency of Nit1 suppresses NSCLCs progression and sensitizes cisplatin treatment response. Continued work is focusing on two parts to elucidate the mechanism of Nit1 deficiency to suppress NSCLCs. One is the impact on apoptosis and cell cycle pathways in lung cancer cells, and the second is the interaction of the immune system and lung cancer cells as Nit1 has been reported as a negative regulator in primary T cells [29–31].

## MATERIALS AND METHODS

### Mouse models and genotyping protocols

All experiments were performed according to protocols approved by the Institutional Animal Care And Use Committee (IACUC) of Thomas Jefferson University and complied with the Guide for the Care and Use of Laboratory Animals. KrasG12D mutated mice were obtained from JAX (Strain Name: 129S/Sv-Kras^tm3Tyj/J^ Stock Number: 008185). They were maintained in a heterozygous state (Kras^G12D/+^) because homozygosity for the Kras LA2 allele is embryonically lethal as described [32]. The Kras^G12D/+^ mice were crossed with Nit1 knockout (Nit1^−/−^) mice, which were provided by Dr. Jianke Zhang [14, 15, 29] to generate Nit1^+/−^:Kras^G12D/+^ F1 mice. Nit1^+/−^:Kras^G12D/+^ were inbred to get F2 Nit1^+/+^:Kras^G12D/+^ and Nit1^−/−^:Kras^G12D/+^ mice, and then backcrossed to the Kras background through at least six generations.

Screening of founder animals and their offspring was performed by genomic PCR with the following primer sets: Nit1 wild-type allele, 5′-GTTGGTCTAGCAATCTGTTATGA-3′ and 5′-GTGCTGGGATTAAAGGTGTGC-3′; Nit1 deletion allele, 5′-GTACCGGATACCGATTACTTCGA-3′ and 5′-GTGCTGGGATTAAAGGTGTGCA-3′; product length: Wild type = 310 bp, Mutant = 250 bp; Kras wild type allele, 5′-TGCACAGCTTAGTGAGACCC-3′ and 5′-GACTGCTCTCTTTCACCTCC-3′; Kras mutant allele, 5′-TGCACAGCTTAGTGAGACCC -3′ and 5′-GGAGCAAAGCTGCTATTGGC-3′; product length: Wild type = 220 bp, Mutant = 390 bp, Heterozygote = 220 bp and 390 bp.

### Western blot analysis

Resected tumor tissue and normal mice lung tissue were subject to a plastic micro tissue homogenizer and sonicated in T-PER tissue protein extraction reagent (Thermo Scientific) with protease and phosphatase inhibitor cocktail, while cultured cell were lysed with M-PER protein extraction reagent. Cell and tissue lysates were centrifuged at 9,000 × g for 10 minutes at 4°C. Supernatants were transferred to clean microcentrifuge tubes, frozen on dry ice, and thawed on ice. Total protein concentrations in the lysates were determined using BCA kit. Equal amount of total proteins (30μg/lane) were loaded on a 10% SDS-PAGE. Membranes were subsequently incubated with various primary antibodies.

### Histologic analysis and immunohistochemistry

Tissue sections were cut at 5 μm and were stained with H&E or immunostained with rabbit anti-mouse Ki67 (1:200; Santa Cruz), followed by the appropriate HRP-conjugated secondary antibodies (1:200; Sigma) and Fast 3,3′-diaminobenzidine (DAB) chromogenic tablets (Sigma). Proliferation was quantified by the expression of Ki67 positive cells (3 images of same size tumors per lung) per microscopic field (×400). Lung and tumor area quantifications were carried out on H&E-stained slides. Pictures of each lung lobe were taken on a Nikon Eclipse 90i microscope. Lung and tumor area were measured using NIS Elements 3.0 SP7 software (Nikon Instruments B.V. Europe). A lung cancer pathologist provided tumor grading and classification according to standard histopathological grading [33].

### Characterization of mouse lung cancer

Paired littermates of F2 (Nit1^+/+^:Kras^G12D/+^ and Nit1^−/−^:Kras^G12D/+^) were sacrificed at different time points ranging from ages 4 to 7 months because these mice develop lung tumors as early as 1 week after birth and do not usually survive >200 days [32]. After preliminary analysis of F2 mice, we sacrificed 4-6 months old Nit1^+/+^:Kras^G12D/+^ and Nit1^−/−^:Kras^G12D/+^ mice that had been backcrossed to S129 background for representative analysis. The lung tissue was immediately removed after the mice were sacrificed and the tumor nodules visible on the lung surface were counted. Tumor diameter was measured with a caliper and tumors were divided into two groups: tumor diameter 0-3 mm (small nodules) and >3 mm (large nodules). The left lungs containing tumors were fixed overnight in 10% neutral buffered formalin. The lungs were then transferred to 70% ethanol and processed for paraffin embedding. Tumor and normal tissue resections of right lung samples were used for protein and total RNA extraction.

### Microcomputed tomography (micro-CT)

We chose 4-6 month old mice to anesthetize with a continuous flow of 1% to 3% isoflurane/oxygen mixture (2 L/min). Non-contrast whole body CT imaging without respiratory or cardiac gating was performed using the MicroCAT II small-animal CT scanner (Siemens ImTek Inc.) for approximately 10 min per mouse. X-rays are generated at 80 kVp and 500 μA. The resulting raw data were converted to the standard DICOM data sets and imported to the MIM6.5 (MIM Software Inc.) for tumor contouring and 3D reconstruction. The image analysis method was based on using threshold and region-grow algorithms to segment the image data and define the separate anatomic structures of interest. Volumes of interest were defined by manually drawing multi-slice regions of interest on the tumor nodules (at 50% of maximum voxels) and the contralateral soft tissue. After the number and volume of nodules were contoured by MIM automatically, the tumor nodule structure was selected and rendered with a different color using the combination of manual segmentation and semi-automated contouring methods. These analyses were consistent between two independent operators and performed by a well-trained researcher in a blinded manner.

### Quantitative real-time RT-PCR

Nit1 mRNA expression was confirmed by quantitative realtime RT-PCR. Total RNAs were amplified with OneStep RTPCR kit (Qiagen) and QuantiTest SYBR Green RT-PCR kit (Qiagen), using primers as follows: 5′-GTACTTTGTACTCAGCCCAG- 3′ (forward) and 5′-CCATAGAGGTCAGGTCTGCG-3′ (reverse). All real-time assays were carried out in triplicate using an Applied Biosystems 7900 HT real-time PCR platform (Life Technologies). The primer set for amplification of glyceraldehyde-3-phosphate dehydrogenase (GAPDH) mRNA served as a housekeeping control gene, and the levels of Nit1 transcripts were determined by the ΔΔCt method [34].

### Tissue microarrays

Using an antibody from Sigma (HPA006657), immunohistochemical staining was conducted to detect Nit1 abundance in human lung cancer and normal tissue microarrays (BC01115b, US BioMax, http://www.biomax.us/tissue-arrays/Lung/BC041115b) according to their standard protocol (http://www.biomax.us/pdfs/ImmunohistochemistryStaining.pdf). The level of Nit1 protein expression was categorized by a semi-quantitative score of the immunostaining intensity by light microscopy evaluation. We used a standard methodology that determined a range of staining intensities from negative to strong with intermediate grades. The intensity of immunoperoxidase staining was scored as 0 (negative), 1 (minimal to low level of positive staining), 2 (moderate expression), or 3 (strong staining). Cores without clear tumor cells were excluded and the final subgroups included: 40 cases of lung squamous cell carcinoma (2 lung adenosquamous carcinomas), 44 lung adenocarcinoma, 4 each of lung bronchioloalveolar carcinoma and large cell carcinoma, 6 small cell undifferentiated carcinoma, 5 carcinoid, plus 10 non-malignant (normal) lung tissue. A pathologist evaluated and scored the cores of the tumors from the patients.

### Cell culture, recombinant adenoviral vector, and siRNA

All human lung cancer cell lines were bought from ATCC and maintained in RMPI1640 supplemented with 10% fetal bovine serum, 100-units/ml penicillin, and 100μg/ml streptomycin (Gibico). Adenoviral recombinant human NIT1 was a gift from Dr. Kay Huebner [14]. All siRNAs targeted to ORF region were bought from Dharmacon and included NIT1 mixture smart pool (L-020022-01-0005), NIT2 smart pool (L-017902-00-0005) and Non-targeting Pool (D-001810-10-20). MTS assay was used for cell viability test.

### Statistical analysis

Data are presented as mean ± SEM. Prism6 software was used for comparison of lung tumor burden between same age Nit1^+/+^:Kras^G12D/+^ and Nit1^−/−^:Kras^G12D/+^ mice. A 2-tailed Mann Whitney test was used unless stated otherwise and P value <0.05 was considered statistically significant.
